# Are changes in attitudes towards school associated with declining youth drinking? A multi-level analysis of 37 countries

**DOI:** 10.1093/eurpub/ckac029

**Published:** 2022-06-01

**Authors:** Abigail K. Stevely, Rakhi Vashishtha, Hannah Fairbrother, Laura Fenton, Madeleine Henney, Michael Livingston, John Holmes

**Affiliations:** 1 Sheffield Alcohol Research Group, School of Health and Related Research (ScHARR), University of Sheffield, UK; 2 Centre for Alcohol Policy Research, La Trobe University, Melbourne, Australia; 3 Health Services and Systems Research, Duke-NUS Medical School, Singapore; 4 Health Sciences School, University of Sheffield, UK; 5 National Drug Research Institute, Curtin University, Perth, Australia; 6 Department of Clinical Neurosciences, Karolinska Institutet, Stockholm, Sweden

**Keywords:** HBSC, Health Behaviour in School-aged Children study, Alcohol Drinking, Underage Drinking, Young people

## Abstract

**Background:**

Changes in adolescents’ attitudes towards school are a potential explanation for recent declines in young people’s alcohol consumption. However, this has not been tested using multi-national survey data, which would permit stronger causal inferences by ruling out other country-specific explanations. This study therefore uses an international survey of schoolchildren to examine associations between changing attitudes towards school and andadolescent alcohol consumption.

**Methods:**

We used data from 247,32515-year-olds across 37 countries participating in four waves of the Health Behaviour in School-aged Children study(2001/02-2013/14). Attitudes towards school were assessed using two measures – self-reported pressure from schoolwork and whether respondents like school. Outcome measures were weekly alcohol consumption and having been drunk twice in one’s lifetime. We used whole population and gender-specific hierarchical linear probability models to assess the relationship between attitudes and alcohol outcomes within countries over time.

**Results:**

Country-level changes over time in liking school were not associated with changes in alcohol consumption. However, a 10% increase in feeling pressured by schoolwork was associated with a 1.8% decline in drunkenness (95%CI: -3.2% - -0.3%) and weakly associated with a 1.7% decline in weekly drinking (95%CI: -3.6% - 0.2%). Among girls only, increases in feeling pressured by schoolwork were associated with a 2.1% decline in weekly drinking (95%CI: -3.7% - -0.6%) and a 2.4% decline in drunkenness (95%CI: -3.8% - -1.1%).

**Conclusion:**

Changes in attitudes towards school may have played a minor role in the decline in alcohol consumption among adolescent girls only.

## Introduction

Over the last two decades, there have been substantial declines in adolescent alcohol consumption across most high-income countries, including much of Europe and North America.^1–5^ Using data collected from 45 countries, the Health Behaviour in School-aged Children (HBSC) study shows a fall in the proportion of 15-year-olds who have been drunk at least twice from 36% in 2001/02 to 20% in 2017/18.^6,7^ This is comparable to the decline seen in similar international school-based surveys, such as the European Schools Project on Alcohol and Drugs (ESPAD).^8^

There is a growing quantitative literature examining proposed explanations for this decline.^3,4,9–11^ In a recent systematic review of studies testing potential explanations, Vashishtha *et al.* found that there is moderate support for changes in parenting as a cause of the decline.^4^ There is also weak evidence that a number of other factors have contributed to the decline, including changes in alcohol policy, exposure to alcohol advertising and declines in face-to-face socialising. However, overall, the existing quantitative literature does not provide conclusive evidence on the drivers of declines in youth drinking.^4^

Changes in attitudes towards school are one posited explanation.^12^ School satisfaction has increased in a number of countries from 2002-2018, particularly among boys,^13^ and children who are more satisfied with their school are less likely to use alcohol and other substances, possibly due to high perceived responsibility for their own learning.^13,14^ Young people (particularly girls ^13^) are also experiencing increased pressure to perform academically in a context of wider economic insecurity following the 2008 financial crisis and other socioeconomic changes.^15–17^ The potential impact of this on youth drinking is unclear. For example, in a recent qualitative study, Caluzzi *et al.* found that health and social wellbeing were important reasons to avoid drinking, with one young woman saying that ‘in order to… achieve things in your life, you don’t want to harm your body in any way. So it makes sense not to drink’.^18^ Conversely, feeling more pressured by schoolwork than your peers may be associated with *heavier* alcohol consumption as a form of stress-relief.^19,20^

Five quantitative studies, all published after the systematic review by Vashishtha *et al.* ^4^, have examined the role of school-related factors in reduced adolescent alcohol consumption over time.^21– 25^ Their findings are mixed, providing some evidence of associations between declines in youth drinking and increased time spent on homework ^23^, reduced truancy ^22,23^, reduced misconduct ^23^. They also include some null or contradictory findings for truancy ^21,25^, academic aspirations ^24^, and attachment to school.^24^ However, this literature is largely focused on Nordic countries. Using data from countries with different levels and time-trends in both attitudes can provide additional insights as it permits stronger causal inference by ruling out explanations pertaining to single countries, such as enforcement of minimum legal drinking age policies or school-based prevention initiatives.^3,26^

This study therefore aims to examine associations between attitudes towards school and adolescent alcohol consumption at the country-level. Since trends over time in attitudes towards school vary by sex, we also examine these associations separately for boys and girls.

## Methods

### Data

HBSC is a repeat cross-sectional international survey of school children. We used data from the four most recent waves of the survey available for general usage at the time of writing – 2001/02, 2005/06, 2009/10 and 2013/14.^27^ HBSC participants self-report information on their socio-demographic characteristics in addition to a range of health-related measures. In each participating country, the study team applied a clustered sampling design, based on a standardised protocol, to select a representative sample such that students were clustered within classes and schools.^28^

HBSC collects data from 11, 13 and 15-year-olds but, as the prevalence of drinking is very low at younger ages, the analytical sample for the present study included only 15-year-olds. We also excluded Albania, Armenia, Bulgaria, Turkey, Greenland and the Republic of Moldova from our analysis as they did not provide data on the measures of interest (school pressure, liking school, drinking frequency, and lifetime frequency of being drunk) for at least three of the four survey waves. The total analytical sample size was therefore 247,325 participants across 37 countries, with a mean of 6,684 participants per country (ranging from 1,666 in Malta to 13,910 in Canada; Supplementary Table 1).

### Measures

#### Attitudes towards school

Our measures of attitudes towards school were *school pressure*, based on the question ‘How pressured do you feel by the schoolwork you have to do?’ with the response options ‘Not at all’, ‘A little’, ‘Some’ and ‘A lot’, and *liking school*, based on the question ‘How do you feel about school at present?’ with the response options ‘I like it a lot’, ‘I like it a bit’, ‘I don’t like it very much’ and ‘I don’t like it at all’. We dichotomised these variables in order to produce a prevalence rate for each country, as required by the statistical approach described below. For the first measure, ‘Some’ and ‘A lot’ indicated feeling pressured by schoolwork. For the second, ‘I like it a lot’ and ‘I like it a bit’ indicated liking school.

#### Alcohol consumption

Our measures of alcohol consumption were *weekly drinking* and *lifetime history of drunkenness.* Participants were classed as drinking at least weekly if they report drinking ‘every week’ or ‘every day’ on any of a series of questions asking how often respondents consumed different alcoholic beverage types (e.g. beer). Questions on beer, wine and spirits consumption were included in all four survey waves, while questions on alcopops (i.e. pre-mixed spirits) were introduced in 2005/06. We included the alcopops measure when present, but also constructed a secondary variable for sensitivity analysis that excludes alcopops.

Participants were also asked to report how many times in their lifetime they have ‘had so much alcohol that you were really drunk’. Our primary analyses used a binary version of this variable, where ‘2-3 times’, ‘4-10 times’ and ‘more than 10 times’ indicate being drunk at least twice in their lifetime. We also retained the full ordinal variable for use in sensitivity analysis.

#### Sex

Since trends in attitudes towards school vary by sex, we included sex-stratified models in our analysis. We also included sex as a control variable in models of the total population. Participants’ sex was measured using the question ‘Are you a girl or a boy?’

### Statistical analysis

We used hierarchical linear probability modelling with robust standard errors to examine the association between changes in attitudes towards school and changes in adolescents’ alcohol consumption. The models partitioned variance in outcome measures across four levels: individuals, schools, years and countries.

We followed the modelling approach described by Fairbrother *et al.* ^26^ and recently applied to study adolescent drinking by Vashishtha *et al*. [Vashishtha R, Holmes J, Pennay A, Deitze PM, Livingston M (forthcoming) ‘An examination of the role of changes in country-level leisure time internet use and computer gaming on adolescent drinking in 33 European countries’, International Journal of Drug Policy.] Our model predictors were survey year (three dummy indicators), participant sex, school pressure, and liking school. The model included three terms for each measure of attitudes towards school: (1) individual participants’ survey responses (2) means for each country (e.g. Austria), and (3) means for each country-year (e.g. Austria in 2001/02). The country-year means were centred by subtracting the country means. The individual-level predictors were centred by subtracting the country-year means.

When interpreting model results, we were primarily interested in the country-year means, which indicate the associations between trends in attitudes towards school and adolescent alcohol consumption. The year dummy variables and country-level means are not testing our hypotheses but are instead controlling for time-varying confounding and differences between countries. Similarly, the individual-level attitudes towards school control for variance arising from differences in the relationship between attitudes towards school and alcohol consumption at the individual and country-level.^29,30^ For example, individuals who feel more pressured than their peers may be more likely to drink alcohol, while increases in the prevalence of feeling pressured by schoolwork over time may be associated with declines in alcohol consumption. Individual-level coefficients reported in the results should be interpreted with caution as the analysis was not designed to produce unbiased estimates at this level.

For the primary analyses we fit six models, three predicting weekly drinking and three predicting drunkenness. For each outcome, we fit a model for the full analytical sample and separate models for girls and boys. We also conducted sensitivity analyses using the alternate weekly drinking variable that excludes consumption of alcopops and the full ordinal measure of drunkenness. All analyses were run on weighted data.

### Ethical approval

This study was approved by the University of Sheffield’s ethics committee and conforms to the principles embodied in the Declaration of Helsinki. Each country that participated in the HBSC study obtained approval to conduct the survey from their ethics review board or equivalent regulatory body. Participation was voluntary, and informed consent (active or passive) was sought from school administrators, parents and children as per national human subject requirements.

## Results

### Descriptive analysis

The prevalence of weekly drinking and drunkenness among 15-year-olds varied widely across the 37 countries included in our analysis. The proportion who reported drinking at least weekly in 2013/14 ranged from 28.8% to 2.6% and the proportion who reported having been drunk at least twice ranged from 38.2% to 6.0% (Table 1). Across all countries between 2001/02 and 2013/14, the average prevalence of weekly drinking fell by 13.4 percentage points (25.6% to 12.2%;), lifetime drunkenness fell by 12.9 percentage points (35.3% to 22.4%), feeling pressure from schoolwork fell by 0.8 percentage points (45.8% to 45.0%) and liking school increased by 5.6 percentage points (63.3% to 68.9%; Table 1).

**Figure 1:**
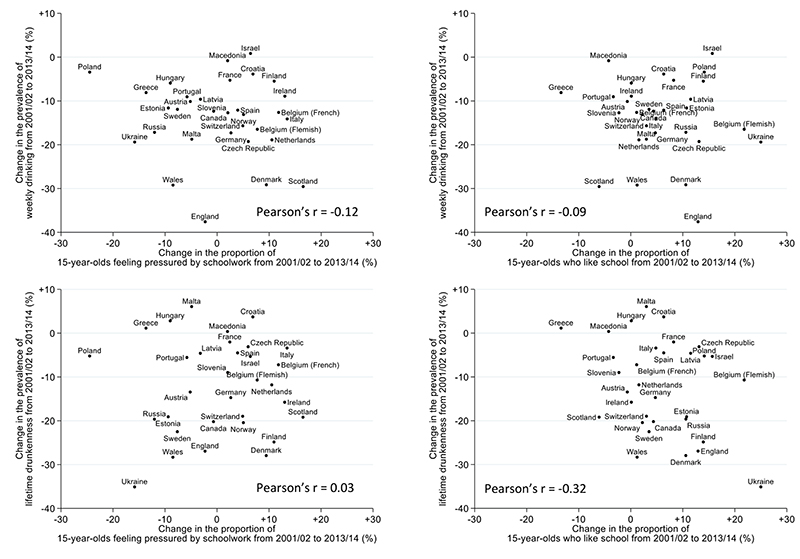
Scatter plots of change from 2001/02 to 2013/14 in school pressure (left) and liking school (right) against change in weekly drinking (top) and lifetime history of drunkenness (drunk at least twice; bottom)

Scatter plots of the country-level change in each school attitude variable and each alcohol consumption variable are shown in Figure 1. These show that the decline in weekly drinking and lifetime drunkenness occurred in most countries. The change in the proportion of adolescents reporting being pressured by schoolwork or liking school was less consistent, with some countries showing increases and others showing decreases.

### Modelling weekly drinking

Table 2 shows the results of the hierarchical linear probability models. A 10% increase over time in school pressure was weakly associated with a 1.7% decrease in weekly drinking (95% CI: -3.6% - 0.2%), while a 10% increase in liking school was weakly associated with a 1.0% decrease in weekly drinking (95% CI:-3.2% - 1.2%). This means that, in the full population model, we did not find strong evidence that country-level changes in attitudes towards school over time explain trends over time in the prevalence of weekly drinking.

Country-level changes in school pressure (β_BOYS_=-1.1%; 95% CI: -3.3% - 1.1%) and liking school (β_BOYS_=-0.7%; 95% CI: -2.8% - 1.3%) over time were also not strongly associated with trends in weekly drinking for boys. In contrast, a 10% increase over time in school pressure within a country was associated with a 2.1% decrease in weekly drinking for girls (95% CI:-3.7% --0.6%), but there was no strong evidence of a relationship between changes in liking school and weekly drinking for girls (β_GIRLS_=-1.1%; 95% CI: -3.3% - 1.0%).

### Modelling having been drunk at least twice

The findings for having been drunk at least twice were broadly consistent with models of weekly drinking (Table 3). A 10% increase in liking school over time within a country was weakly associated with a 1.2% decrease in lifetime prevalence of being drunk at least twice (95% CI: -3.6% - 1.2%), but a 10% increase in school pressure was associated with a 1.8% decrease in the drunkenness outcome (95% CI: -3.2% - -0.3%; Table 3).

Sex-stratified models suggest that girls drove the association between country-level changes over time in school pressure and drunkenness. A 10% higher prevalence of feeling pressured by schoolwork among girls was associated with a 2.4% decrease in the lifetime prevalence of having been drunk at least twice (95% CI: -3.8% - -1.1%). In contrast, for boys, the decrease was smaller and the confidence interval was wider (β_BOYS_=-0.8%; 95% CI: -2.3% - 0.7%).

### Sensitivity analyses

The results of sensitivity analyses using the alternate weekly drinking variable excluding consumption of alcopops and the full ordinal measure of lifetime drunkenness were consistent with the primary analyses (Supplementary Tables 2 & 3).

## Discussion

The results above show that changes in attitudes towards school were not consistently associated with changes in alcohol consumption during a period of declining adolescent alcohol consumption. For boys, there was no clear evidence that changes in a country’s prevalence of liking school or feeling pressured by schoolwork were associated with changes in alcohol consumption measures. However, for girls, increases in a country’s prevalence of feeling pressured by schoolwork were associated with decreases in the prevalence of both weekly drinking and having been drunk at least twice in their lifetime. This association was seen only for feeling pressured by schoolwork and not for liking school.

The implications of these findings for understanding the role of school pressure in the decline in youth drinking depend on trends in school pressure. A large *increase* in the proportion of young people feeling pressured by school would suggest it has played a potentially important role in the decline. Although the descriptive analysis of HBSC data reported above finds that the average prevalence of feeling school pressure slightly decreased, this average change is the result of divergence across countries – in 19 of the 37 countries there were *increases* in the prevalence of feeling school pressure, of up to 17%. In recent analysis of the HBSC data, Löfstedt *et al.* found that school pressure increased among girls between 2002 and 2018 in 20 of the 32 included North American and European countries.^13^ This suggests that changes in school pressure have played a minor role in the decline in youth drinking overall, but may have made larger contributions in countries where school pressure increased over time and particularly for girls. These findings on the effect of changing school pressure over time among girls differ from previous literature examining the effects of stressors on adolescent alcohol consumption. Grigsby *et al.* and Liu *et al.* find that work stress and stressful life events are associated with increased alcohol use and negative consequences of drinking.^19,31^ This may reflect a difference between the effects of pressure from schoolwork and other stressors among adolescent girls.

Our analysis of the HBSC survey included a large and nationally representative sample of adolescents across 37 countries from 2001/02 to 2013/14. The survey included measures of attitudes towards school and alcohol consumption that were consistent across countries and over time. The multi-level modelling approach used also enabled us to test hypotheses regarding associations at the country-level over time, while accounting for the clustering of participants within schools, years, and countries. Although our measures of attitudes towards school and alcohol consumption may be subject to self-report biases, the HBSC survey uses an internationally standardised questionnaire which students self-complete in the classroom. The study does however have limitations. Firstly, the relationships between attitudes towards school and alcohol consumption outcomes may have shifted since the 2013/14 survey wave, and during the COVID-19 pandemic. Secondly, we classed anyone drinking one beverage type at least weekly as a weekly drinker. This method may underestimate the prevalence of weekly drinking by missing some adolescents who do not drink any single beverage type every week but do drinking some type of alcohol every week. Furthermore, the measures of attitudes towards school are challenging to interpret, as adolescents may report liking school or feeling pressured by school for a range of reasons. For example, some adolescents may like school because they are happy with their life overall, while others may like school as it offers a space away from difficult family conditions. To further our understanding of the relationship between attitudes towards school and alcohol consumption, future research could consider causal links between the HBSC measures and broader life satisfaction or pressures such as home instability. Further analysis could also consider variation in the magnitude of the decline in youth drinking by socio-economic status. Researchers in England have found some evidence that the group of young people who drink have become more likely to receive free school meals (a proxy of socio-economic status) over time, which may have implications for health inequalities.^32^

This study contributes to the growing quantitative evidence base examining potential explanations for the decline in youth drinking. We found that changes over time in girls’ attitudes towards school may have played a minor role in the decline in adolescent alcohol consumption, but do not explain the full magnitude of the decline. This is rapidly becoming a theme in studies on the topic, where there is a lack of strong evidence across multiple potential domains of explanation, suggesting that there is no single dominant driver of trends in youth drinking.^3,4^ Future research should explore the potential for more complex processes of change, driven by multiple intersecting trends. In particular, it may be useful to consider the changing position of alcohol consumption in wider youth culture and in relation to other health behaviours, since there are concurrent declines in other risky behaviours such as illicit drug use and smoking cigarettes.^33,34^

## Supplementary Material

Supplementary material

## Figures and Tables

**Table 1:** Summary statistics on the proportion of 15-year-olds who drink weekly, have been drunk at least twice, feel pressured by schoolwork and like school

Variable	Mean across all included countries	Standard deviation	Range
**Prevalence of weekly** **drinking**			
All time points	20.1%	0.08	6.1% - 39.5%
2001/02	25.6%	0.10	10.5% - 47.6%
2005/06	24.2%	0.11	8.0% - 51.5%
2009/10	19.8%	0.09	5.9% - 37.3%
2013/14	12.2%	0.06	2.6% - 28.8%
**Lifetime prevalence of being** **drunk at least twice**			
All time points	30.0%	0.10	13.9% - 54.7%
2001/02	35.3%	0.13	11.2% - 66.2%
2005/06	32.7%	0.11	15.1% - 57.3%
2009/10	31.0%	0.11	13.6% - 55.3%
2013/14	22.4%	0.08	6.0% - 38.2%
**Prevalence of feeling** **pressured by schoolwork**			
All time points	44.8%	0.13	25.1% - 74.0%
2001/02	45.8%	0.15	22.5% - 78.8%
2005/06	45.3%	0.13	23.4% - 67.6%
2009/10	42.2%	0.13	18.4% - 67.0%
2013/14	45.0%	0.15	18.5% - 73.9%
**Prevalence of liking** **school**			
All time points	67.0%	0.10	46.7% - 90.3%
2001/02	63.3%	0.11	42.6% - 86.0%
2005/06	66.8%	0.11	48.1% - 88.9%
2009/10	68.6%	0.11	46.6% - 90.6%
2013/14	68.9%	0.11	46.1% - 90.7%

**Table 2:** Results from models showing associations between weekly drinking, feeling pressured by schoolwork, and liking school

	Full model			Girls only			Boys only		
	B	95% CI		B	95% CI		B	95% CI	
***Year***									
2001/02 (ref)	-	-	-	-	-	-	-	-	-
2005/06	-0.003	-0.028	0.021	0.010	-0.016	0.036	-0.016	-0.042	0.010
2009/10	-0.043	-0.074	-0.012	-0.026	-0.056	0.004	-0.061	-0.096	-0.025
2013/14	-0.122	-0.151	-0.093	-0.101	-0.127	-0.075	-0.144	-0.181	-0.106
***Sex***									
Boy (ref)	-	-	-	-	-	-	-	-	-
Girl	-0.079	-0.093	-0.065	-	-	-	-	-	-
***Individual*** ***school attitudes***									
Pressured by schoolwork	0.014	0.007	0.021	0.012	0.005	0.019	0.013	0.006	0.021
Like school	-0.092	-0.102	-0.083	-0.088	-0.098	-0.077	-0.096	-0.108	-0.085
***Country mean*** ***school attitudes***									
Pressured by schoolwork	-0.070	-0.301	0.162	-0.021	-0.202	0.160	-0.124	-0.410	0.163
Like school	-0.072	-0.284	0.140	-0.123	-0.314	0.068	-0.030	-0.274	0.214
***Country-year*** ***mean school*** ***attitudes***									
Pressured by schoolwork	-0.169	-0.362	0.023	-0.214	-0.366	-0.061	-0.111	-0.327	0.105
Like school	-0.101	-0.319	0.116	-0.113	-0.330	0.104	-0.074	-0.275	0.127

**Table 3:** Results from models showing associations between lifetime frequency of being drunk, feeling pressured by schoolwork, and liking school

	Full model			Girls only			Boys only		
	B	95% CI		B	95% CI		B	95% CI	
***Year***									
2001/02 (ref)	-	-	-	-	-	-	-	-	-
2005/06	-0.011	-0.034	0.011	-0.003	-0.025	0.018	-0.020	-0.046	0.005
2009/10	-0.029	-0.056	-0.002	-0.018	-0.044	0.009	-0.044	-0.074	-0.013
2013/14	-0.111	-0.144	-0.078	-0.092	-0.127	-0.056	-0.131	-0.166	-0.097
***Sex***									
Boy (ref)	-	-	-	-	-	-	-	-	-
Girl	-0.045	-0.063	-0.026	-	-	-	-	-	-
***Individual*** ***school attitudes***									
Pressured by schoolwork	0.022	0.013	0.032	0.020	0.011	0.030	0.021	0.012	0.031
Like school	-0.135	-0.145	-0.124	-0.139	-0.151	-0.126	-0.130	-0.140	-0.119
***Country mean*** ***school attitudes***									
Pressured by schoolwork	0.010	-0.213	0.232	0.066	-0.139	0.271	-0.033	-0.288	0.223
Like school	0.139	-0.135	0.414	0.119	-0.186	0.425	0.132	-0.148	0.412
***Country-year*** ***mean school*** ***attitudes***									
Pressured by schoolwork	-0.177	-0.320	-0.033	-0.242	-0.376	-0.107	-0.079	-0.228	0.070
Like school	-0.119	-0.361	0.122	-0.075	-0.351	0.201	-0.133	-0.352	0.085

## Data Availability

No new data were generated or analysed in support of this research.

## Key Points

- This study uses a large scale multi-national school survey to provide the most comprehensive examination to date of the role of school attitudes in declining youth drinking.
- Changes over time in the proportion of 15-year-olds who like school are not associated with changes in alcohol consumption.
- Increases over time in the proportion of 15-year-olds who feel pressured by schoolwork is associated with a small decline in youth drinking, particularly for girls.
- Overall, changes in attitudes towards school may have played a minor role in the decline in youth drinking.
