# Supplementary material for: Are changes in attitudes towards school associated with declining youth drinking? A multi-level analysis of 37 countries
Source: Eur J Public Health. Author manuscript; Available in PMC 2022 Jun 6. (PMC9159339; doi:10.1093/eurpub/ckac029)
Supplement: Supplementary material [file EMS143853-supplement-Supplementary_material.pdf]

Supplementary Table 1. Number of participants in each country included in analyses

**Countries included in analyses**	**Sample size**				
	**Total**	**2001/2**	**2005/6**	**2009/10**	**2013/14**
All countries	247325	50576	64099	68580	64070
Austria	5876	1298	1494	1820	1264
Belgium (Flemish)	6589	2030	1616	1226	1717
Belgium (French)	6068	1381	1414	1341	1932
Canada	13910	1207	2289	5441	4973
Croatia	7446	1446	1630	2424	1946
Czech Republic	6607	1660	1665	1522	1760
Denmark	5421	1380	1552	1226	1263
Estonia	5521	1267	1587	1398	1269
Finland	7505	1745	1685	2110	1965
France	8482	2614	2222	1906	1740
Germany	8045	1749	2552	1640	2104
Greece	5708	1324	1416	1648	1320
Hungary	5350	1330	1187	1733	1100
Iceland	8879	0	1883	3680	3316
Ireland	5819	919	1685	1695	1520
Israel	6780	1567	1997	1352	1864
Italy	5372	1229	1335	1546	1262
Latvia	5548	1117	1330	1375	1726
Lithuania	5558	1905	1861	1792	0
Luxembourg	3968	0	1507	1382	1079
Malta	1666	667	354	0	645
Netherlands	5450	1273	1363	1457	1357
Norway	5467	1624	1534	1339	970
Poland	7333	2152	2287	1410	1484
Portugal	5098	802	1383	1553	1360
Romania	5049	0	1605	2002	1442
Russia	8621	2575	2754	1847	1445
Slovakia	5001	0	1252	1914	1835
Slovenia	6060	1069	1561	1815	1615
Spain	10583	1756	3065	2003	3759
Sweden	7608	1226	1526	2090	2766
Switzerland	7498	1540	1500	2246	2212
Ukraine	7021	1601	1829	1897	1694
Macedonia	6301	1412	1896	1536	1457
England	5950	1773	1451	1118	1608
Scotland	7783	1149	2198	2567	1869
Wales	5583	1164	1350	1637	1432
USA	4801	1625	1284	1892	0

Supplementary Table 2. Results from sensitivity analyses showing associations between weekly drinking (excluding alcopops), feeling pressured by schoolwork, and liking school

Full model			
	B	95% CI	
Year			
2001/02 (ref)	-	-	-
2005/06	-0.036	-0.059	-0.012
2009/10	-0.065	-0.097	-0.034
2013/14	-0.135	-0.165	-0.105
Sex			
Boy (ref)	-	-	-
Girl	-0.089	-0.103	-0.074
Individual school attitudes			
Pressured by schoolwork	0.013	0.006	0.020
Like school	-0.087	-0.097	-0.077
Country mean school attitudes			
Pressured by schoolwork	-0.063	-0.281	0.156
Like school	-0.053	-0.250	0.143
Country-year mean school attitudes			
Pressured by schoolwork	-0.126	-0.317	0.065
Like school	-0.105	-0.329	0.119
**Girls only**			
	**B**	**95% CI**	
Year			
2001/02 (ref)	-	-	-
2005/06	-0.033	-0.057	-0.009
2009/10	-0.056	-0.086	-0.027
2013/14	-0.117	-0.144	-0.090
Sex			
Boy (ref)	-	-	-
Girl	-	-	-
Individual school attitudes			
Pressured by schoolwork	0.012	0.006	0.018
Like school	-0.081	-0.091	-0.070

Country mean school attitudes			
Pressured by schoolwork	-0.016	-0.181	0.149
Like school	-0.099	-0.266	0.068
Country-year mean school attitudes			
Pressured by schoolwork	-0.151	-0.294	-0.008
Like school	-0.116	-0.334	0.102
**Boys only**			
	**B**	**95% CI**	
Year			
2001/02 (ref)	-	-	-
2005/06	-0.038	-0.065	-0.012
2009/10	-0.076	-0.112	-0.039
2013/14	-0.154	-0.192	-0.116
Sex			
Boy (ref)	-	-	-
Girl	-	-	-
Individual school attitudes			
Pressured by schoolwork	0.012	0.004	0.020
Like school	-0.093	-0.104	-0.082
Country mean school attitudes			
Pressured by schoolwork	-0.114	-0.392	0.164
Like school	-0.012	-0.245	0.222
Country-year mean school attitudes			
Pressured by schoolwork	-0.087	-0.308	0.134
Like school	-0.076	-0.286	0.134

Supplementary Table 3. Results from sensitivity analyses showing associations between lifetime frequency of being drunk (full ordinal scale), feeling pressured by schoolwork, and liking school

	Full model		
	**B**	**95% CI**	
Year			
2001/02 (ref)	-	-	-
2005/06	-0.039	-0.106	0.028
2009/10	-0.101	-0.185	-0.018
2013/14	-0.360	-0.463	-0.257
Sex			
Boy (ref)	-	-	-
Girl	-0.161	-0.218	-0.103
Individual school attitudes			
Pressured by schoolwork	0.071	0.044	0.099
Like school	-0.433	-0.470	-0.395
Country mean school attitudes			
Pressured by schoolwork	0.019	-0.684	0.722
Like school	0.408	-0.458	1.275
Country-year mean school attitudes			
Pressured by schoolwork	-0.635	-1.069	-0.201
Like school	-0.252	-0.930	0.426
